# A CRISPR-Assisted Nonhomologous End-Joining Strategy for Efficient Genome Editing in Mycobacterium tuberculosis

**DOI:** 10.1128/mBio.02364-19

**Published:** 2020-01-28

**Authors:** Mei-Yi Yan, Si-Shang Li, Xin-Yuan Ding, Xiao-Peng Guo, Qi Jin, Yi-Cheng Sun

**Affiliations:** a MOH Key Laboratory of Systems Biology of Pathogens, Institute of Pathogen Biology, and Center for Tuberculosis Research, Chinese Academy of Medical Sciences and Peking Union Medical College, Beijing, China; b Sanming Project of Medicine in Shenzhen on Construction of Novel Systematic Network against Tuberculosis, National Clinical Research Center for Infectious Diseases, Shenzhen Third People’s Hospital, Southern University of Science and Technology, Shenzhen, China; University of Pittsburgh

**Keywords:** CRISPR-Cas system, *Mycobacterium marinum*, *Mycobacterium smegmatis*, *Mycobacterium tuberculosis*, genome editing, nonhomologous end joining

## Abstract

New tools for genetic manipulation of Mycobacterium tuberculosis are needed for the development of new drug regimens and vaccines aimed at curing tuberculosis infections. Clustered regularly interspaced short palindromic repeat (CRISPR)–CRISPR-associated protein (Cas) systems generate a highly specific double-strand break at the target site that can be repaired via nonhomologous end joining (NHEJ), resulting in the desired genome alteration. In this study, we first improved the NHEJ repair pathway and developed a CRISPR-Cas-mediated genome-editing method that allowed us to generate markerless deletion in Mycobacterium smegmatis, Mycobacterium marinum, and M. tuberculosis. Then, we demonstrated that this system could efficiently achieve simultaneous generation of double mutations and large-scale genetic mutations in M. tuberculosis. Finally, we showed that the strategy we developed can also be used to facilitate genome editing in Escherichia coli.

## Abstract (precis)

The global health impact of M. tuberculosis necessitates the development of new genetic tools for its manipulation, to facilitate the identification and characterization of novel drug targets and vaccine candidates. Clustered regularly interspaced short palindromic repeat (CRISPR)–CRISPR-associated protein (Cas) genome editing has proven to be a powerful genetic tool in various organisms; to date, however, attempts to use this approach in M. tuberculosis have failed. Here, we describe a genome-editing tool based on CRISPR cleavage and the nonhomologous end-joining (NHEJ) repair pathway that can efficiently generate deletion mutants in M. tuberculosis. More importantly, this system can generate simultaneous double mutations and large-scale genetic mutations in this species. We anticipate that this CRISPR-NHEJ-assisted genome-editing system will be broadly useful for research on mycobacteria, vaccine development, and drug target profiling.

## INTRODUCTION

Mycobacterium tuberculosis, the infectious agent of tuberculosis (TB), caused 10 million infections and 1.57 million deaths in 2017 (1). The global health impact of M. tuberculosis necessitates the development of genetic tools for manipulating M. tuberculosis to identify and characterize appropriate drug targets and vaccine candidates. Genetic approaches using nonreplicating vectors, long linear DNA fragments, recombineering, and specialized phage transduction have been developed to manipulate M. tuberculosis (2–8). Recently, the ORBIT method, which combines oligonucleotide-mediated recombineering and Bxb1 integrase targeting, was developed for the creation of mutants with large numbers of deletions, insertions, or fusions in the bacterial chromosome (9). However, these approaches usually yield low numbers of mutants and require multiple steps to generate markerless mutants. The generation of a single markerless mutation can require months, while the generation of multiple mutations, which is usually required for the development of live bacterial vaccines and functional studies of redundant genes, can require years. In addition, the current methods usually leave one scar from the generation of one markerless mutation (9, 10), which may be problematic when multiple mutations must be introduced. More importantly, recombination-mediated chromosomal rearrangements or deletions might occur between these scars, possibly hindering the use of live bacterial vaccines bearing multiple scars (11).

Clustered regularly interspaced short palindromic repeat (CRISPR)–CRISPR-associated protein (Cas) systems, including CRISPR–CRISPR-associated protein 9 (Cas9) and CRISPR-Cas12a (Cpf1), have been widely used as genome-editing tools (12, 13). The CRISPR-Cas9 system generates a Cas9-mediated double-strand break (DSB) guided either by two small RNAs, i.e., a CRISPR RNA (crRNA) and a *trans*-acting crRNA (tracrRNA), or by a chimeric single guide RNA (sgRNA) (14, 15). In contrast, Cas12a, which is a type V-A endonuclease of the class 2 CRISPR-Cas system, is a dual nuclease involved in both crRNA processing and DNA cleavage guided by a single crRNA without the need for a tracrRNA (16). Recognition and cleavage of target DNA by CRISPR-Cas requires a short DNA sequence next to the protospacer, the protospacer adjacent motif (PAM) (17). The DNA breaks can be repaired either via a homologous recombination (HR)-mediated repair pathway or via the nonhomologous end-joining (NHEJ) repair pathway, leading to precise or imprecise genome editing, respectively (18, 19). The NHEJ pathway is error prone and repairs the DSB by generating insertions and/or deletions at the cleavage site that disrupt the targeted gene. Unlike HR-mediated repair pathways, the NHEJ repair pathway does not require a homologous DNA template, which simplifies genome-editing procedures (18). Recently, HR- and NHEJ-assisted CRISPR genome-editing approaches have been developed in many bacteria (20–23), including Mycobacterium smegmatis (24, 25); however, none of these approaches can be used in M. tuberculosis.

The NHEJ pathway plays a major role in DSB repair in eukaryotic cells (26, 27), whereas the HR-mediated pathway is more critical for DSB repair in bacterial cells (28–30). The HR-mediated and NHEJ pathways might compete with each other in bacteria and mammalian cells (29, 31–33). Mutation of RecA, a major player in the HR-mediated pathway, strongly induces the NHEJ repair pathway in M. smegmatis (31). In contrast to the many components required for NHEJ repair in eukaryotic organisms, the bacterial NHEJ pathway requires only two key components: Ku and multifunctional ligase D (LigD) (28, 34–36). The Ku protein binds to the DNA ends and recruits LigD, which then processes and ligates the DNA ends (37). Mycobacterial NHEJ is the most studied bacterial NHEJ system (28, 38, 39); however, it is far from being completely understood. The additional ATP-dependent DNA ligase LigC1 can compensate for loss of LigD ligase activity, suggesting that this NHEJ pathway might be more complex than the basic two-component system (40).

**FIG 1:**
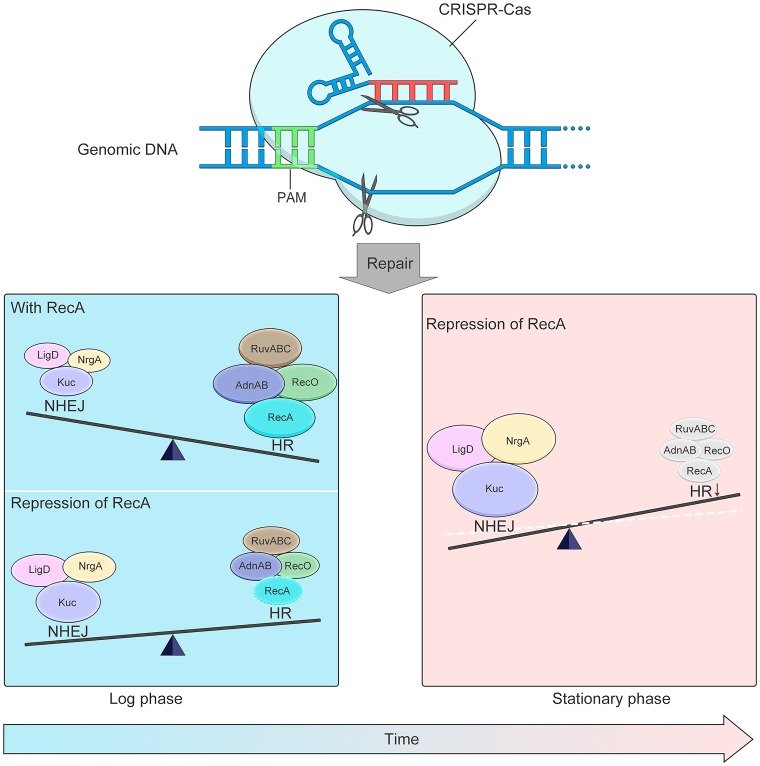
Cartoon representation of CRISPR-Cas–NHEJ-assisted genome editing in mycobacteria. Genome editing is completed in two steps: (i) cleavage by the CRISPR-Cas system and (ii) NHEJ-mediated repair. High expression of the MmNHEJ machinery (Ku, LigD, and NrgA from M. marinum) facilitates efficient genome editing in M. marinum (bottom left, “With RecA”). Repression of RecA-dependent homologous recombination (HR) can increase the efficiency of NHEJ, thereby increasing genome-editing efficiency in M. smegmatis (bottom left, “Repression of RecA”). NHEJ efficiency is higher when double-strand breaks are generated during stationary phase; thus, efficient genome editing can be achieved in RecA-repressed M. tuberculosis cells in stationary phase (bottom right).

In this study, we made three changes to increase the activity of the NHEJ repair pathway (Fig. 1): we (i) increased the expression of Mycobacterium marinum NHEJ machinery (MmNHEJ, comprising Ku, LigD, and NrgA, the product of a previously uncharacterized gene we designated *nrgA*), (ii) repressed RecA-dependent HR, and (iii) generated DSBs in stationary phase. By applying these strategies for promoting NHEJ together with CRISPR-Cas cleavage, we developed an efficient method for robust, template-independent genome editing in M. smegmatis, M. marinum, and M. tuberculosis (Fig. 1).

## RESULTS

### CRISPR-NHEJ-mediated genome editing in M. marinum.

**FIG 2:**
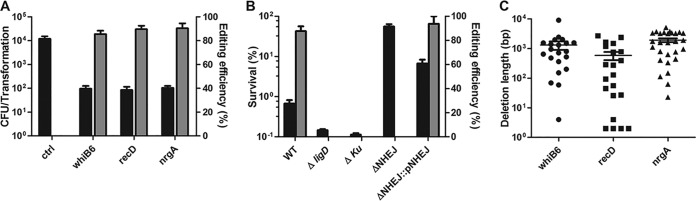
CRISPR-Cas12a-assisted NHEJ genome editing in M. marinum. (A) Genome-editing efficiency in the presence of CRISPR-Cas12a cleavage in M. marinum. Plasmids pYC1103, pYC1178, and pYC1521, expressing *whiB6*, *recD*, and *nrgA*-targeting crRNA, respectively, were transformed into M. marinum with the Cas12a-expressing plasmid pMV261-Cas12a. (B) Genome-editing efficiency of *whiB6* with CRISPR-Cas12a cleavage in wild-type M. marinum and its derivatives. The *whiB6* crRNA plasmid (pYC1103) was transformed into the wild type and its derivatives. Transformation efficiency was defined as the total number of CFU generated per transformation. Editing efficiency was calculated as the ratio of the number of edited events to the total number of colonies analyzed by PCR and sequencing. Survival was calculated by comparing the number of transformants to the number of control transformants carrying the empty vector. (A, B) Bars represent mean values ± standard deviations from two independent experiments. (C) Deletion length distribution of the indicated genes resulting from CRISPR-Cas12-assisted genome editing. Bars represent the median deletion size for each strain.

We previously reported a Cas12a-assisted recombineering system that allowed precise genetic manipulation in M. smegmatis (24). By coupling CRISPR-Cas12a cleavage with the recombineering mediated by the mycobacteriophage recombination enzyme Che9c, it is possible to achieve highly efficient genome editing using single-stranded DNA (ssDNA) or double-stranded DNA (dsDNA) as a repair template (24). However, this system does not work in M. tuberculosis (data not shown). To investigate whether this system functions in other mycobacteria, we transplanted it into M. marinum, a slowly growing mycobacterium similar to M. tuberculosis, to modify the nonessential gene *whiB6*. Compared with the outcome for the no-cleavage control, CRISPR-Cas12a cleavage killed most of the bacteria, resulting in survival of approximately 1% of the transformants (Fig. S1A in the supplemental material). Interestingly, the surviving cells were not precisely modified via HR as anticipated; they instead carried deletion mutations (Fig. S1B). Further investigation showed that formation of this CRISPR-Cas12a cleavage-mediated genome modification was independent of the presence of the Che9c system (Fig. 2A) but required Ku and LigD (Fig. 2B), suggesting that it occurred via the NHEJ repair pathway. Consistent with the observation that a *ku* and *ligD* double mutant M. smegmatis strain did not simply phenocopy *ligD* and *ku* single mutant strains (31), deletion of the *ku ligD* locus in M. marinum eliminated NHEJ repair but increased resistance to Cas12a cleavage (Fig. 2B). Complementation of the mutation using a plasmid containing the *ku ligD* locus with the native promoter decreased transformant survival but increased NHEJ repair efficiency (Fig. 2B). Sequencing analysis showed that the survivors that lacked the desired mutations were not susceptible to Cas12a-mediated killing due to complete or partial deletion of the Cas12a-encoding gene. We further determined the deletion sizes via amplification of the repair junctions in the genome-edited strains. The sequence analysis revealed that random deletions occurred around the cleavage site and that the deletion sizes ranged from 2 bp to 10,179 bp (Fig. 2C). Whole-genome sequencing revealed two to three single-nucleotide variants (SNVs) that were apparently not caused by off-target effects in six independent M. marinum mutants, suggesting that the genome editing mediated by this method is specific (Table S1).

10.1128/mBio.02364-19.1 FIG S1 CRISPR-Cas12a-assisted genome editing in M. marinum. (A) Transformation efficiency resulting from electroporation of *whiB6* crRNA plasmids and templates into M. marinum expressing the recombinase and Cas12a. For genome editing, the *whiB6* crRNA plasmid (pYC1103) was transformed with or without repair DNA template (oligonucleotide or dsDNA) into M. marinum harboring plasmid pJV53-Cas12a. Transformation efficiency was defined as the total number of CFUs obtained per transformation. Bars represent the mean ± standard deviation of at least two independent experiments. (B) Molecular outcomes of CRISPR-Cas12a-assisted genome editing. The two halves of the Cas12a cleavage site are shown in red and blue. Uppercase and underlined sequences represent the PAM. Upstream and downstream sequences represent the junction sequences upstream and downstream of the cleavage site. Size of deletion represents the number of nucleotides resected from either end. Download FIG S1, TIF file, 2.6 MB. Copyright © 2020 Yan et al. 2020 Yan et al. This content is distributed under the terms of the Creative Commons Attribution 4.0 International license.

10.1128/mBio.02364-19.7 TABLE S1 Nonspecific mutations identified via whole-genome sequencing. (A) The original laboratory and the edited strains were subjected to whole-genome sequencing. Sequence coverage was calculated as the summed base pairs of the mapped sequence divided by 6,660,144 bp for the M. marinum strain M genome sequence or 4,419,977 bp for the M. tuberculosis strain H37Ra genome sequence. Nonspecific mutations are listed in Table S1B. (B) The SNVs or indels detected via whole-genome sequencing as in Table S1A. Download Table S1, PDF file, 0.1 MB. Copyright © 2020 Yan et al. 2020 Yan et al. This content is distributed under the terms of the Creative Commons Attribution 4.0 International license.

**FIG 3:**
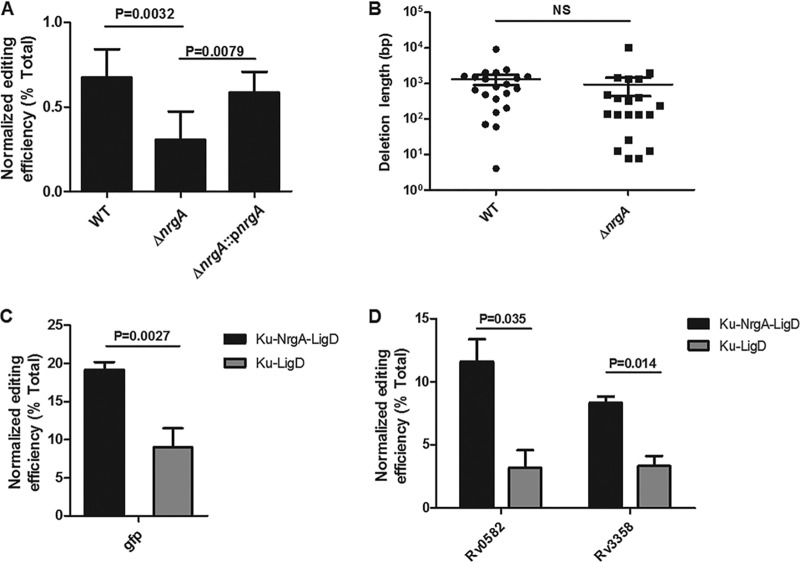
NrgA is involved in CRISPR-NHEJ genome editing in mycobacteria. (A) Genome-editing efficiency in M. marinum derivatives. The *whiB6* crRNA plasmid (pYC1103) was transformed into the wild-type, *nrgA* mutant, or *nrgA*-complemented strain, and 24 colonies in each group were picked for PCR and sequencing analysis. Editing efficiency was calculated as the ratio of the number of edited events to the total number of colonies tested by PCR. Normalized editing efficiency was calculated as the editing efficiency × (total CFU obtained with *whiB6* targeting sgRNA/total CFU obtained with control sgRNA). (B) Deletion length distributions of *whiB6* gene in wild-type and *nrgA* mutant strains in one experiment. Bars represent median deletion size for each strain. NS, not significant. (C, D) NrgA increases the CRISPR-NHEJ genome-editing efficiency in M. smegmatis (C) and M. tuberculosis (D). crRNA plasmids were electroporated into M. smegmatis and M. tuberculosis cells with plasmids expressing the complete NHEJ machinery (pNHEJ-Cas12a-*recX* for M. smegmatis and pNHEJ-*recX* for M. tuberculosis; Ku-NrgA-LigD) or the NHEJ machinery without NrgA (pYC1376 for M. smegmatis and pYC1654 for M. tuberculosis; Ku-LigD). For M. smegmatis, a chromosomally integrated *gfp* reporter gene was edited. Normalized efficiency was calculated as the frequency of GFP-negative (white) transformants × (total CFU obtained with *gfp*-targeting sgRNA/total CFU obtained with control sgRNA). For M. tuberculosis, 24 colonies in each group were picked for PCR and sequencing analysis. Normalized editing efficiency was calculated as the editing efficiency × (total CFU obtained with target sgRNA/total CFU obtained with control sgRNA). (A, C, D) Bars represent mean values ± standard deviations from three independent experiments. *P* values were determined via Student’s unpaired *t* test.

A gene belonging to the phosphofructokinase B-type (PfkB) family of sugar kinase-encoding genes (MMAR_4574), which we named *nrgA* (NHEJ-related gene A), lies between the Ku and ligase D genes in the M. marinum chromosome (Fig. S2A). *nrgA* is conserved in many mycobacteria, and it is usually associated with *ku* and *ligD* (Fig. S2A); however, it is absent in M. tuberculosis. Mutation of *nrgA* significantly decreased the genome-editing efficiency but did not affect the deletion size (Fig. 3), suggesting that *nrgA* might play an important role in the M. marinum NHEJ repair pathway.

10.1128/mBio.02364-19.2 FIG S2 (A) Genomic organization of the *nrgA* (*pfkB*) genes of various mycobacteria. This schematic is taken from the PubSEED web site (R. Overbeek, T. Begley, R. M. Butler, J. V. Choudhuri, et al., Nucleic Acids Res 33:5691–5702, 2005, https://doi.org/10.1093/nar/gki866). (B) Schematic diagram of four different Cas9_Sth1_-sgRNA orientations in the M. tuberculosis RD1 region. Download FIG S2, TIF file, 2.3 MB. Copyright © 2020 Yan et al. 2020 Yan et al. This content is distributed under the terms of the Creative Commons Attribution 4.0 International license.

### CRISPR-NHEJ-mediated genome editing in M. smegmatis.

**FIG 4:**
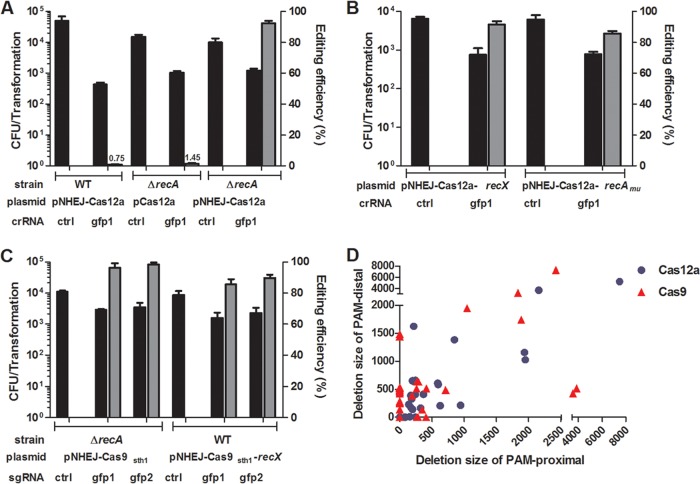
CRISPR-NHEJ-assisted genome editing in M. smegmatis. (A) CRISPR-Cas12a–NHEJ genome-editing efficiency in M. smegmatis derivatives. The *gfp* crRNA plasmid was transformed into wild-type and *recA* mutant strains harboring various plasmids. (B) RecX or RecA_mu_ expression mimicked *recA* deletion in M. smegmatis. Transformation and *gfp*-editing efficiency resulted from electroporation of the indicated *gfp* crRNA plasmids into M. smegmatis expressing the MmNHEJ machinery, Cas12a, and RecX (or RecA_mu_). (C) CRISPR-Cas9_Sth1_-assisted NHEJ genome editing in M. smegmatis. The *gfp* sgRNA plasmid was transformed into *recA* mutant and wild-type strains containing the indicated plasmids. Bars represent mean values ± standard deviations from three independent experiments. (D) Comparison of the Cas9_Sth1_- and Cas12a-induced deletion sizes in PAM-proximal and PAM-distal regions. Transformation efficiency was defined as the total number of CFU obtained per transformation, and editing efficiency was calculated by determining the proportion of GFP-negative colonies.

NHEJ-mediated genome editing could not be achieved in M. smegmatis using Cas12a nuclease in our previous study (24). To increase NHEJ repair efficiency, we overexpressed MmNHEJ using a plasmid containing the *ku ligD* locus from M. marinum in an M. smegmatis strain carrying a chromosomally integrated green fluorescent protein (GFP) reporter (24) and then tested for genome-editing efficiency by monitoring for loss of the GFP signal. To our disappointment, only 0.75% of the transformants were mutated even when the MmNHEJ machinery was expressed (Fig. 4A). As there is a dynamic interplay between HR- and NHEJ-mediated DSB repair (31), we hypothesized that inhibiting HR might stimulate NHEJ-mediated genome editing. To test this hypothesis, we examined the NHEJ-mediated genome-editing efficiency in a *recA* null strain. Approximately 1.45% of the transformants were genome edited in the *recA* null background, whereas more than 90% of the transformants were genome edited when MmNHEJ was expressed in the *recA* null background (Fig. 4A).

To facilitate NHEJ-mediated genome editing in various strain backgrounds, we sought to construct a system to limit RecA function without disrupting its gene. A dominant-negative mutant RecA with an R60C mutation (RecA_mu_ bearing a change of R to C at position 60) can interact with wild-type RecA monomers to inhibit RecA activity in Escherichia coli (41, 42). In addition, RecX negatively regulates RecA activity by blocking RecA assembly onto ssDNA tracts (43–45) or by facilitating more rapid RecA filament disassembly (46, 47). We constructed new plasmids carrying M. smegmatis RecA_mu_ or RecX together with MmNHEJ and Cas12a (Fig. S3D and E) and tested their effects on genome-editing efficiency. The expression of RecA_mu_ or RecX showed phenotypes similar to that of *recA* deletion, i.e., enhanced NHEJ-mediated genome-editing efficiency (Fig. 4B). Furthermore, Cas9_Sth1_ (Cas9 from Streptococcus thermophilus), a Cas9 orthologue developed for regulated gene silencing in mycobacteria (48), can also be used for genome editing in M. smegmatis (Fig. 4C). Compared with the outcomes with the no-cleavage control, CRISPR-Cas12a- and Cas9_Sth1_-assisted NHEJ genome editing usually resulted in the survival of approximately 20% of the transformants, of which more than 80% were genome edited (Fig. 4B and C). Investigation of the end-joining patterns at the cleavage junctions showed that Cas9_Sth1_- and Cas12a-assisted editing typically resulted in random deletions around the cleavage site that ranged from 1 to more than 10 kb in length (Fig. 4D).

10.1128/mBio.02364-19.3 FIG S3 Maps of the plasmids developed for CRISPR-NHEJ-assisted genome editing in M. smegmatis. (A) pNHEJ-Cas12a. (B) pNHEJ-Cas9. (C) pYC1655. (D) pNHEJ-Cas12a-*recX*. (E) pNHEJ-Cas12a-*recA*_mu_. (See also Table S4.) Download FIG S3, TIF file, 1.7 MB. Copyright © 2020 Yan et al. 2020 Yan et al. This content is distributed under the terms of the Creative Commons Attribution 4.0 International license.

### CRISPR-NHEJ-mediated genome editing in M. tuberculosis.

**FIG 5:**
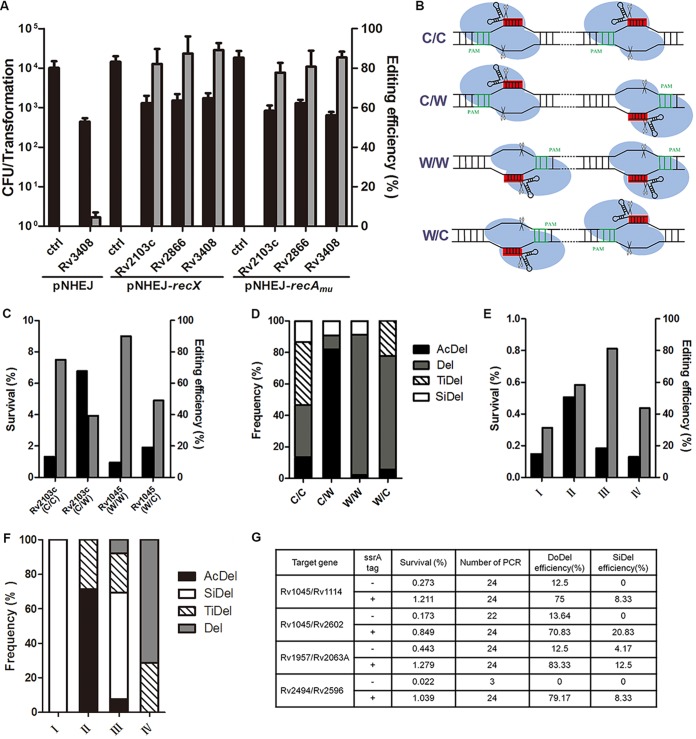
CRISPR-NHEJ-assisted genome editing in M. tuberculosis. (A) CRISPR-Cas9_Sth1_ cleavage combined with NHEJ repair leads to efficient genome editing in M. tuberculosis H37Ra. sgRNA-expressing plasmids were electroporated into M. tuberculosis cells harboring various NHEJ-expressing plasmids. Transformation efficiency was defined as the total number of CFU obtained per transformation, and editing efficiency was calculated as the ratio of the number of edited events to the total number of colonies tested. Bars represent mean values ± standard deviations from two independent experiments. (B to D) Analysis of NHEJ efficiencies induced by paired Cas9_Sth1_-sgRNAs in four different orientations. Four different Cas9_Sth1_-sgRNA orientations were guided by paired PAMs. C/C, C/W, W/W, and W/C orientations were defined by the positioning of the paired PAMs on either the Watson strand (W) or the Crick strand (C). (E, F) Analysis of editing efficiency induced by paired Cas9_Sth1_-sgRNAs at the RD1 regions (I, II, III, and IV represent four different Cas9_Sth1_-sgRNA orientations guided by paired PAMs; see Fig. S2B). (G) Simultaneous generation of double mutations in M. tuberculosis using paired sgRNAs with Cas9_Sth1_ or Cas9_Sth1_-*ssrA*. Survival was defined as the ratio of the number of CFU obtained from the indicated sgRNA to the number of CFU obtained from the control plasmids, and editing efficiency was calculated as the ratio of the number of edited events to the total number of colonies tested; at least 32 colonies were analyzed by PCR and sequenced for each group. DoDel, double deletion. (D, F) The frequencies of accurate deletion (AcDel), single deletion (SiDel), two individual deletions (TiDel), and deletion (Del) were calculated as the ratios of the number of events from each group to the total number of edited events.

As CRISPR-assisted NHEJ genome editing is highly efficient in M. marinum and M. smegmatis, we sought to edit the M. tuberculosis genome using this system. However, neither Cas9- nor Cas12a-assisted genome editing functioned when the plasmid system described above was tested in M. tuberculosis (data not shown). Further analysis showed that the Cas12a cleavage activity might be too weak for it to be useful as an editing tool in M. tuberculosis (Fig. S4). In contrast, the Cas9_Sth1_ cleavage activity is too strong, and leaky Cas9_Sth1_ expression results in DSB generation and cell death (Fig. S4). Given that NHEJ activity is induced in stationary phase (49, 50), we speculated that delaying CRISPR-Cas expression to allow DSB formation only during stationary phase might increase genome-editing efficiency. To test this prediction, we constructed a new two-plasmid cotransformation system in which one plasmid encodes the MmNHEJ machinery and M. tuberculosis RecX/RecA_mu_ (pNHEJ-*recX*-*sacB* and pNHEJ-*recA_mu_*-*sacB*) and the other encodes Cas9 and the sgRNA (pYC1640 or pYC2085) (Fig. S5). To simplify the procedure for curing the helper plasmids, we introduced a *sacB* cassette into the NHEJ-expressing plasmid and used a plasmid vector containing the pMF1 replicon (51), which is unstable in mycobacteria, for expression of Cas9 and the sgRNA (Fig. S5). Three genes were chosen as editing targets, and 10% of transformants were obtained relative to the number obtained with the vector control strain, more than 80% of which were genome edited (Fig. 5A). Molecular analysis of the targeting loci in these transformants revealed that most transformants carried small PAM-distal deletions exactly 3 bp upstream from the PAM sequence that ranged from 1 to 324 bp in length, while a few transformants carried insertions (Fig. S6). In addition, sequences with weak PAMs can also be efficiently edited using our system (Table S2). To test for a possible nonspecific mutator effect of this system, 6 independent M. tuberculosis mutants were chosen for whole-genome sequencing. The results showed that no mutations or at most one mutation was introduced during the genome editing (Table S1).

10.1128/mBio.02364-19.4 FIG S4 Transformation efficiency resulting from electroporation of the Rv3408 sgRNA plasmids into M. tuberculosis expressing Cas9_Sth1_ or Cas12a. The transformation efficiency was defined as the total number of CFUs obtained per transformation. The bars represent the mean ± standard deviation of at least two independent experiments. Download FIG S4, TIF file, 0.8 MB. Copyright © 2020 Yan et al. 2020 Yan et al. This content is distributed under the terms of the Creative Commons Attribution 4.0 International license.

10.1128/mBio.02364-19.5 FIG S5 Maps of the plasmids developed for CRISPR-NHEJ-assisted genome editing in M. tuberculosis. (A) pNHEJ-*recX*-*sacB*. (B) pNHEJ-*recA*_mu_-*sacB*. (C) pYC1640. (D) pYC2085. (E) pYC1446. (See also Table S4.) Download FIG S5, TIF file, 1.7 MB. Copyright © 2020 Yan et al. 2020 Yan et al. This content is distributed under the terms of the Creative Commons Attribution 4.0 International license.

10.1128/mBio.02364-19.6 FIG S6 Molecular outcomes of CRISPR-Cas9_Sth1_-assisted NHEJ in M. tuberculosis with overexpression of mutant RecA or RecX. The frequencies of identical junctions and the numbers of deleted or inserted nucleotides are indicated. The PAM sequences are underlined. The targeted sequences are shown in red, and the inserted sequences are shown in bold capital letters. Download FIG S6, TIF file, 2.7 MB. Copyright © 2020 Yan et al. 2020 Yan et al. This content is distributed under the terms of the Creative Commons Attribution 4.0 International license.

10.1128/mBio.02364-19.8 TABLE S2 Genome editing of regions with weak PAM sequences in M. tuberculosis. Download Table S2, PDF file, 0.2 MB. Copyright © 2020 Yan et al. 2020 Yan et al. This content is distributed under the terms of the Creative Commons Attribution 4.0 International license.

To explore the possibility of using this system to create larger deletions in M. tuberculosis, we used paired sgRNAs with different PAM configurations to generate large deletions within a gene (Fig. 5B). Approximately 0.95% to 6.77% transformants were obtained relative to the number obtained with the vector control strain, among which the editing was more efficient when the PAM sequences were located in a combination of the Watson and Watson strands (W/W) (Fig. 5C). Upon investigation of the end-joining patterns at the cleavage junctions, we noticed that accurate NHEJ (two Cas9 cleavage sites were directly ligated, leading to accurate deletion) was more efficient when the PAM sequences were located in a combination of the Crick and Watson strands (C/W) (Fig. 5D). Next, we successfully deleted the region of difference 1 (RD1) region (Fig. 5E and F), which is more than 9 kb long and contains nine genes (Fig. S2B).

Given that double mutations could be generated when two sgRNAs were used for RD1 region deletion (Fig. 5F), we hypothesized that this system could be used to simultaneously generate double mutations at different genomic sites in M. tuberculosis. Confirming this hypothesis, the desired double mutations could be generated, but at a low efficiency (Fig. 5G). The addition of an *ssrA* tag, a 13-amino-acid sequence appended to the C terminus of Cas9 that decreases leaky expression by promoting proteolytic degradation of the tagged nuclease (52), dramatically increased the genome-editing efficiency for generation of the double mutant (Fig. 5G). To test whether this system can be used for multiple rounds of genome editing, we sequentially mutated four genes (*Rv3408*, *Rv0059*, *Rv2494*, and *Rv2596*) in M. tuberculosis H37Ra. Finally, we showed that this system can be used for high-efficiency genome editing in M. tuberculosis H37Rv and in Mycobacterium bovis BCG (Table S3).

10.1128/mBio.02364-19.9 TABLE S3 Genome editing in M. tuberculosis H37Rv and M. bovis BCG. Download Table S3, PDF file, 0.04 MB. Copyright © 2020 Yan et al. 2020 Yan et al. This content is distributed under the terms of the Creative Commons Attribution 4.0 International license.

### Investigation of the roles of toxins in M. tuberculosis.

**FIG 6:**
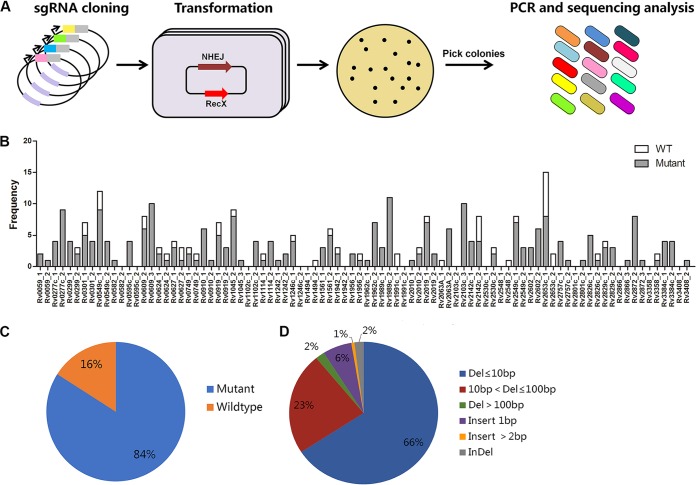
Construction of the toxin gene knockout library. (A) Schematic overview of the construction of the CRISPR knockout toxin gene library. (B) Numbers of colonies with different sgRNAs detected. Edited colonies are shown in gray, and nonedited colonies are shown in white. (C) Genome-editing efficiency in the knockout library. Editing efficiency was calculated as the ratio of the number of edited events to the total number of colonies tested. (D) Frequencies of the indicated mutation events among the mutants detected.

Given the high efficiency of this system, it could be used for pooled knockout (KO) library construction. As a proof of concept, we sought to construct a mutant library of toxin-antitoxin (TA) systems. Endonuclease-deficient Cas9_Sth1_ (dCas9_Sth1_) has been constructed in an L5-integrating plasmid for use in CRISPR interference (CRISPRi) in mycobacteria (48). We modified this plasmid by exchanging dCas9_Sth1_ with Cas9_Sth1_, resulting in plasmid pYC1446 (Fig. S5D). We further designed 88 sgRNAs to target 44 toxin genes, including 43 whose products are toxic when expressed in M. smegmatis and *vapC5* (*Rv0627*), which is an essential toxin gene predicted based on transposon library screening (53). These sgRNAs were constructed in the integrating plasmid pYC1446, and then the resulting plasmids were mixed and transformed into M. tuberculosis strain H37Ra harboring pNHEJ-*recX*-*sacB* (Fig. 6A), yielding approximately 3 × 10^4^ transformants. Three hundred one transformants were picked for PCR and sequencing analysis, among which 84% carried at least one mutation in one of 41 toxin genes (Fig. 6B and C). Similar to the individually generated mutants (Fig. S6), most of the mutants generated in this pool contained deletions of less than 10 bp (Fig. 6D). In addition, contrary to a previous report that *vapC5* is an essential gene (53), this experiment yielded three *vapC5* mutants using two sgRNAs (Fig. 6B), suggesting that *vapC5* is not an essential gene.

### RecA repression improves CRISPR-NHEJ-mediated genome editing in E. coli.

**FIG 7:**
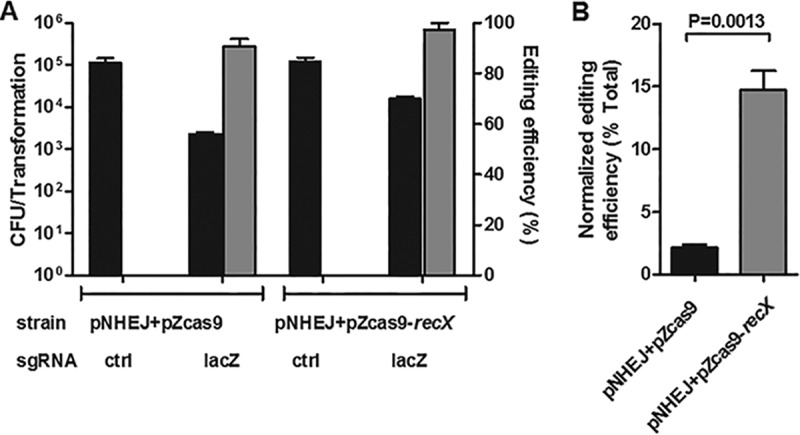
RecA repression improves CRISPR-NHEJ genome editing in E. coli. (A) Transformation and CRISPR-NHEJ genome-editing efficiency obtained by genome editing in E. coli with or without RecX overexpression. Plasmid psgRNA-*lacZ* was transformed into E. coli MG1655 harboring pNHEJ and pZCas9 (or pZCas9-*recX*). Editing efficiency was calculated as the ratio of the number of edited events (i.e., white colonies on the X-Gal plate) to the total number of transformants. (B) Normalized editing efficiency was calculated as the ratio of edited events normalized against the transformation efficiency. Bars represent the mean values ± standard deviations from three independent experiments. For normalized editing efficiency, *P* values were determined by Student’s unpaired *t* test.

Cas9 has been used with NHEJ repair for genome editing in prokaryotes and archaea, such as E. coli and Methanosarcina acetivorans (23, 54); however, the genome-editing efficiency in these bacteria is low. RecA has homologues in many organisms, suggesting that inhibiting RecA activity might improve the efficiency of NHEJ-mediated repair. To test this hypothesis, we took advantage of a Cas9-NHEJ genome-editing system that has been used to generate gene deletions in E. coli (23). RecX overexpression resulted in a more-than-5-fold increase in the efficiency of Cas9-NHEJ-mediated genome editing of the *lacZ* gene in E. coli (Fig. 7). This result suggests that the strategy we have developed for CRISPR-NHEJ-mediated genome editing in mycobacteria can be used in other organisms.

## DISCUSSION

The system developed in this study will have a transformative impact on the speed, scale, and scope of research that can be accomplished in mycobacteria. First, our system represents the most efficient and convenient method for generating gene deletions in mycobacteria. Current methods, such as ORBIT (9), need the experimenter to be skilled in the preparation of highly efficient competent cells in order to obtain the desired mutants. With our method, edited transformants are obtained at a rate of about 10% of the transformant control (Fig. 5A), making the technique easily used for all experimenters. In addition, precise deletion of defined length in target genes can be obtained via the use of paired sgRNAs. Moreover, our method can easily be used for generation of gene deletions in M. marinum, which is difficult to genetically manipulate using current methods. Second, genome editing using this system does not leave any scars, which is important for vaccine development. Furthermore, following genome editing, the bacteria can be easily cured of the helper plasmids in this system. Third, this system can be used to simultaneously generate double mutations, which cannot be achieved by the current methods, allowing us to generate *N* clean mutations in M. tuberculosis within *N* + 2 months rather than the normal requirement for 4*N* or more months using traditional methods. This trait is especially important when working with this slowly growing mycobacterium, in which the generation of multiple mutations is prohibitively time consuming. Finally, this system is scalable and can be used for genome screening. Genomewide screens with CRISPRi technology have been widely used in many organisms, including prokaryotes and eukaryotes (55–57), whereas CRISPR KO screening has only been used in eukaryotes due to the low efficiency of NHEJ-mediated genome editing in prokaryotes. Given the high efficiency of our system, which allowed more than 10^4^ mutants to be obtained from one transformation, the generation of large mutant libraries for CRISPR KO screening is feasible. Moreover, each sgRNA sequence functions as a barcode, allowing quantification of each knockout strain in the pool using next-generation sequencing. Based on these features, the system we have described here adds a key tool for simple construction of M. tuberculosis mutants that might facilitate the study of mycobacterial biology, the construction of potential live M. tuberculosis vaccines, and the identification of essential drug targets.

NHEJ is the predominant DSB repair pathway in eukaryotes, and it depends on multiple components, including the Ku70/Ku80 heterodimer, polymerases, nucleases, and ligases (26, 27). Furthermore, multiple accessory proteins might be involved in cleaning the DSB ends to make them suitable for repair (27). In contrast, the bacterial NHEJ system was thought to consist of only two components until LigC1 was shown to promote NHEJ in a strain harboring a point mutation in the LigD ligase domain (LigD-LIG) (40). Here, we showed that NrgA might be also involved in NHEJ repair in mycobacteria. This finding is supported by the following two pieces of evidence. First, *nrgA* is closely linked to the genes encoding Ku and LigD in many mycobacteria (Fig. S2A in the supplemental material), and second, NrgA expression increased the genome-editing efficiency 2- to 3-fold in M. marinum, M. smegmatis, and M. tuberculosis (Fig. 3). NrgA might act in concert with Ku and LigD to facilitate the DSB repair, which might mirror the NHEJ system in eukaryotes, in which multiple accessory proteins are involved in DSB repair. Furthermore, additional factors might also be involved in the NHEJ repair pathway in mycobacteria, based on the observation that the deletion sizes generated via NHEJ repair differ in M. tuberculosis, M. smegmatis, and M. marinum (Fig. 2C and 4D; Fig. S6). The factors involved in the NHEJ repair pathway, and their roles, remain to be elucidated.

RecA-dependent HR is the preferred DSB repair pathway in prokaryotes (42), and the NHEJ pathway is inhibited in order to favor HR when possible (i.e., when a repair template is available) (42). However, the dynamic relationship between NHEJ and HR is bidirectional, as repression of RecA-dependent HR also stimulates the NHEJ pathway (Fig. 4A) (31). Given that RecA has homologues in every kingdom of life (42, 58), repression of RecA might improve the efficiency of NHEJ-mediated genome editing in other organisms, including prokaryotes and archaea. Thus, the paradigm of the CRISPR-NHEJ system we developed in this study could be adapted for genome editing to facilitate research on other organisms.

## MATERIALS AND METHODS

### Strains, media, and growth conditions.

E. coli strain MG1655, M. marinum strain M, M. bovis BCG, M. tuberculosis strains H37Ra and H37Rv, M. smegmatis strain mc^2^155, and their derivatives were used in this study. Unless otherwise indicated, M. tuberculosis H37Ra was used. M. smegmatis was grown in Middlebrook 7H9 broth (Difco) supplemented with 0.05% Tween 80 and 0.2% glycerol or on Middlebrook 7H10 plates supplemented with the appropriate antibiotics. M. marinum, M. bovis BCG, and M. tuberculosis were grown in Middlebrook 7H9 broth (Difco) supplemented with 0.05% Tween 80, 0.2% glycerol, and oleic acid-albumin-dextrose-catalase (OADC; Becton Dickinson) or on 7H10 plates supplemented with the appropriate antibiotics, 0.5% glycerol, and OADC (Becton Dickinson). When indicated, antibiotics and small molecules were used at the following concentrations: kanamycin (25 μg/ml), hygromycin (50 μg/ml), zeocin (50 μg/ml), and anhydrotetracycline (ATc) (50 ng/ml).

### Plasmids.

pMV261-Cas12a and pCR-Hyg (or pCR-Zeo) were used in M. marinum. The genes encoding the MmNHEJ machinery (*MMAR_457*3, *MMAR_4574*, and *MMAR_4575*) were amplified from the M. marinum chromosome. Plasmids containing *cas9*_Sth1_ were originally obtained from pLJR965 (48). pNHEJ-Cas12a-*recX* and pNHEJ-Cas12a-*recA*_mu_ (or pNHEJ-Cas9_Sth1_-*recX*) were used in M. smegmatis, and pYC1655 was constructed to express the cognate sgRNA. pNHEJ-*recX-sacB* (or pNHEJ-*recA*_mu_-*sacB*) were used in M. tuberculosis. A codon-optimized *cas9* gene under the control of the TetR-regulated promoter and the sgRNA cassette under the control of an optimized TetR-regulated promoter were cloned into a plasmid harboring the pMF1 replicon (51) to yield pYC1640 (or pYC2085). The sgRNA cassette contains two BbsI restriction sites for insertion of the target sequence. A *recX* allele was amplified from the E. coli chromosome and cloned into pZCas9 (23) to create pZCas9-*recX*. All plasmids used in this study are described in Table S4 in the supplemental material. Details of plasmid constructions are available upon request.

10.1128/mBio.02364-19.10 TABLE S4 Plasmids used in this study. Download Table S4, PDF file, 0.2 MB. Copyright © 2020 Yan et al. 2020 Yan et al. This content is distributed under the terms of the Creative Commons Attribution 4.0 International license.

### Genome editing in M. smegmatis.

Cells harboring pNHEJ-Cas12a-*recX* (or pNHEJ-Cas9_Sth1_-*recX*) were grown in 4 ml of 7H9 medium supplemented with 0.05% Tween 80, 0.2% glycerol, and 25 μg/ml kanamycin. One or two milliliters of starter culture was used to inoculate 100 ml complete 7H9 broth in a 250-ml flask, which was incubated at 37°C overnight. Competent M. smegmatis cells were prepared as previously described (59). About 300 ng of the crRNA-expressing plasmid was mixed with the competent cells. Electroporation was performed with the following settings: 2.5 kV, 25 μF, and 1,000-Ω resistance. After electroporation, 1 ml of 7H9 broth was added to the cells, which were then immediately incubated for 4 h at 30°C. Next, the cultures were plated on 7H10 agar supplemented with the appropriate antibiotics and 50 ng/ml ATc. When targeting the *gfp* gene, the editing efficiency was calculated as the frequency of GFP-negative (white) transformants. Each time, at least 24 GFP-negative colonies were picked for PCR and sequencing analysis. To cure the helper plasmids, a single colony was picked and grown to saturation in 7H9 medium at 37°C, followed by plating on 7H10 plates. The colonies from these plates were streaked to screen for loss of antibiotic resistance, and more than 70% of the colonies had lost the two helper plasmids in most cases.

### Genome editing in M. marinum.

Cells harboring pMV261-Cas12a were grown in 4 ml of 7H9 medium supplemented with 0.05% Tween 80, 0.2% glycerol, OADC, and 25 μg/ml kanamycin. One or two milliliters of starter culture was used to inoculate 100 ml complete 7H9 broth in a 250-ml flask, which was incubated at 30°C for 3 to 5 days. Competent M. marinum cells were prepared as previously described (60). Electroporation of M. marinum was performed in a similar manner as for M. smegmatis. After electroporation, the cells were cultured in 1 ml of 7H9 broth supplemented with OADC and incubated overnight at 30°C. Next, the cultures were plated on 7H10 agar supplemented with OADC, the appropriate antibiotics, and 50 ng/ml ATc. The plates were incubated for 10 to 15 days at 30°C, after which time transformants were picked for PCR and sequencing analysis. Target-specific primers were designed to amplify sequences at least 1,000 bp upstream and downstream from the chromosomal sequences flanking the cleavage sites. The PCR products were analyzed via agarose gel electrophoresis, and the colonies with smaller or no PCR products were regarded as mutants. The PCR products amplified from the transformants with sizes similar to that of the wild-type strain were further analyzed via sequencing. The editing efficiency was calculated as the ratio of the number of edited events to the total number of colonies analyzed by PCR and sequencing. To cure the helper plasmids, a single colony was picked and grown to saturation in 7H9 medium supplemented with OADC at 30°C, followed by plating the cells on 7H10 plates supplemented with OADC. The colonies from these plates were streaked to screen for loss of antibiotic resistance, and more than 50% of the colonies had lost the two helper plasmids in most cases.

### Genome editing in M. tuberculosis.

Cells harboring pNHEJ-*recX*-*sacB* (or pNHEJ-*recA*_mu_-*sacB*) were grown in 10 ml of 7H9 medium supplemented with 0.05% Tween 80, 0.2% glycerol, OADC, and 25 μg/ml kanamycin. One or two milliliters of starter culture was used to inoculate 100 ml complete 7H9 broth in a roller bottle, which was incubated at 37°C for 5 to 7 days. At an optical density at 600 nm (OD_600_) of ∼0.8, 10 ml of 15% sterilized glycine stock solution was added, yielding a final concentration of 1.5%, and the incubation continued at 37°C with rolling for an additional 20 to 24 h. Competent M. bovis BCG and M. tuberculosis cells were prepared as previously described (61). Electroporation of M. bovis BCG and M. tuberculosis was performed similarly to that of M. marinum and M. smegmatis with the following modifications. After electroporation, the cells were cultured in 1 ml of 7H9 broth supplemented with OADC and incubated for 2 days at 37°C to allow cells to grow at high density, mimicking the stationary-phase environment. Next, the cultures were plated on 7H10 agar supplemented with OADC, the appropriate antibiotics, and 50 ng/ml ATc. The plates were incubated for 20 to 30 days at 37°C, and transformants were then picked for PCR and sequencing analysis. To cure the helper plasmids, a single colony was picked and grown to saturation in 7H9 medium supplemented with OADC and 5 μg/ml kanamycin at 37°C. The cells were then diluted 1:100 in 1 ml of 7H9 medium supplemented with OADC and grown for 5 days. The cultures were diluted and plated on 7H10 plates supplemented with OADC and 2% sucrose. The colonies from these plates were streaked to screen for loss of antibiotic resistance, and more than 70% of the colonies had lost the two helper plasmids in most cases.

For the sequential mutation of four genes in M. tuberculosis, the *Rv3408* mutant was first constructed as described above using a Zeo^r^ sgRNA-expressing plasmid. Next, a verified colony was picked to prepare competent cells, which were then transformed with a Hyg^r^ sgRNA-expressing plasmid targeting *Rv0059*. The transformants obtained were verified to have the *Rv0059* mutation and loss of Zeocin resistance. A verified colony was picked to prepare competent cells, which were then transformed with a Zeo^r^ plasmid expressing double sgRNAs targeting *Rv2494* and *Rv2596* to simultaneously generate the double mutations, generating an M. tuberculosis strain with mutations in four genes.

### Genome editing in E. coli.

Genome editing in E. coli was performed as described previously (23). Briefly, the competent E. coli cells harboring the appropriate plasmids were transformed with control or *lacZ* targeting the sgRNA plasmid and then plated on the appropriate selective LB plate supplemented with X-Gal (5-bromo-4-chloro-3-indolyl-β-d-galactopyranoside) (40 μg/ml) and IPTG (isopropyl-β-d-thiogalactopyranoside) (100 μM). Editing efficiency was calculated as the ratio of white colonies on the X-Gal plate to the total number of transformants. Normalized editing efficiency was calculated as the editing efficiency × (total CFU obtained with *lacZ*-targeting sgRNA/total CFU obtained with control sgRNA).

### Whole-genome sequencing.

Edited strains and strain H37Ra harboring the helper plasmids pNHEJ-*recX* or pNHEJ-*recA*_mu_ were incubated at 37°C until they reached an OD_600_ of 0.6 to 0.8. M. marinum and M. tuberculosis chromosomal DNA was extracted as previously described (62) and then sonicated into fragments of less than 500 bp. The fragments were treated with End Prep enzyme mixture for end repair, 5′-end phosphorylation, and dA tailing in one reaction, followed by a T-A ligation reaction to add adaptors to both ends. Size selection of the adaptor-ligated DNA was then performed using the AxyPrep Mag PCR clean-up kit (Axygen), and fragments of approximately 410 bp (with an approximate insert size of 350 bp) were recovered. Each sample was then amplified via PCR for eight cycles using the P5 and P7 primers, both of which carry sequences that can anneal with the flow cell to perform bridge PCR. The P7 primer also carries a 6-base index that allows multiplexing. The PCR products were cleaned up using the AxyPrep Mag PCR clean-up kit (Axygen), validated using an Agilent 2100 Bioanalyzer (Agilent Technologies, Palo Alto, CA, USA), and quantified with a Qubit2.0 fluorometer (Invitrogen, Carlsbad, CA, USA). Sequencing was carried out using a 2 × 150-bp read length, and approximately 500-fold coverage for the genome size was expected.

### Construction of the CRISPR knockout library.

A total of 88 sgRNA-expressing plasmids targeting 44 toxin genes were constructed individually. Purified pooled plasmids were transformed into M. tuberculosis H37Ra cells carrying pNHEJ-*recX*-*sacB*. The resulting transformants were first picked for analysis of the transformed sgRNAs and then for verification of the genome editing via PCR and sequencing analysis.

### Data availability.

The data that support the findings of this study are available from the corresponding authors upon request. Short-read data in this study have been deposited at the NCBI Sequence Read Archive (SRA) with the accession number PRJNA559662.
