# Supplementary figures and images for: A CRISPR-Assisted Nonhomologous End-Joining Strategy for Efficient Genome Editing in Mycobacterium tuberculosis
Source: mBio. 2020 Jan 28;11(1):e02364-19. doi: 10.1128/mBio.02364-19 (PMC6989103; doi:10.1128/mBio.02364-19)

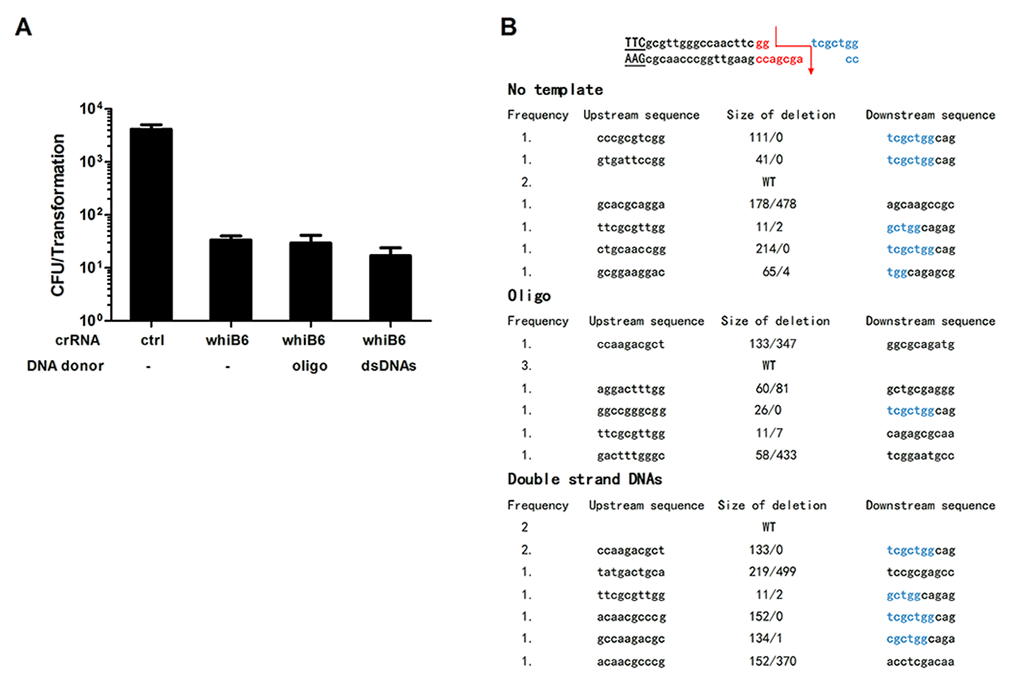

Supplement: FIG S1 [file mBio.02364-19-sf001.tif]

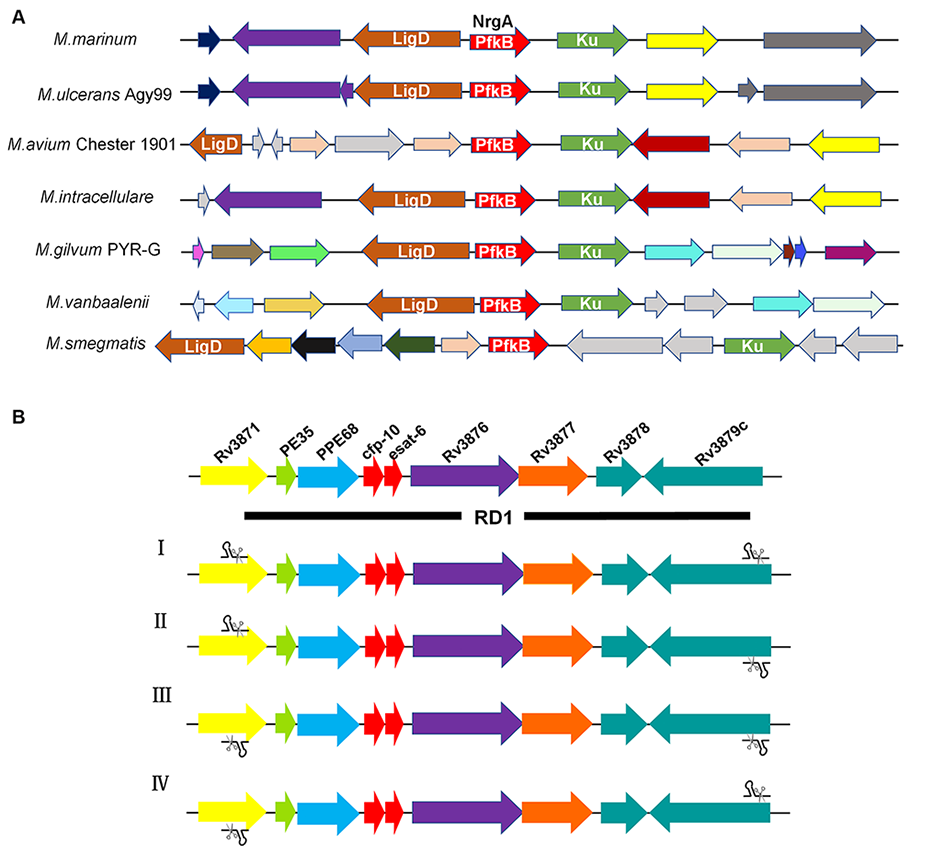

Supplement: FIG S2 [file mBio.02364-19-sf002.tif]

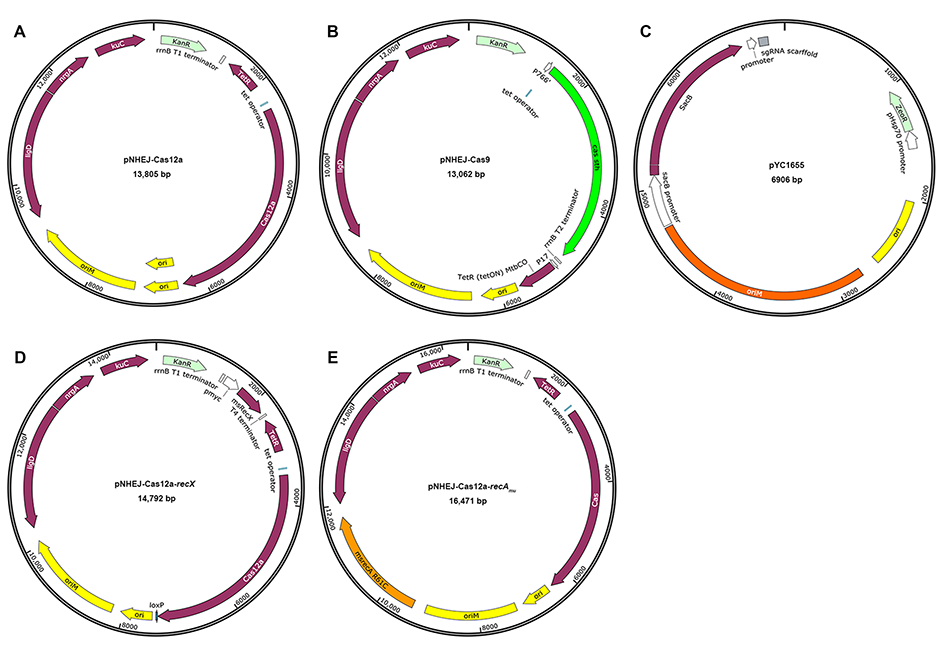

Supplement: FIG S3 [file mBio.02364-19-sf003.tif]

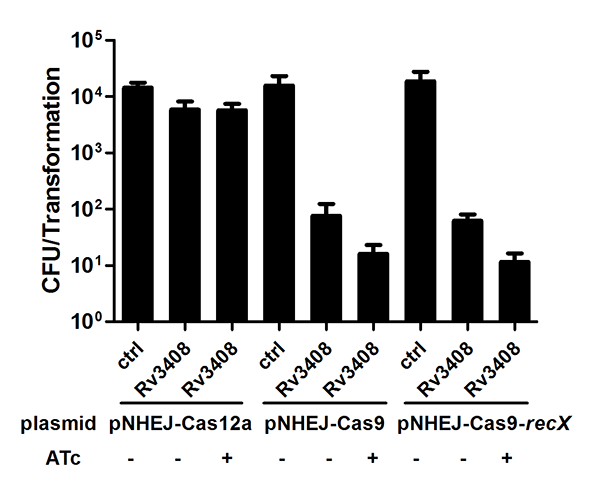

Supplement: FIG S4 [file mBio.02364-19-sf004.tif]

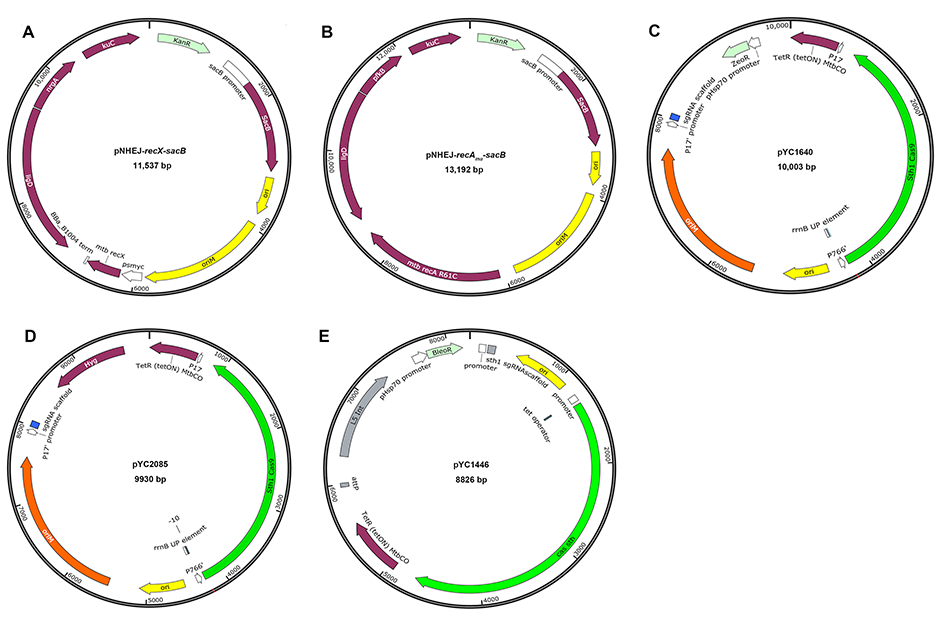

Supplement: FIG S5 [file mBio.02364-19-sf005.tif]

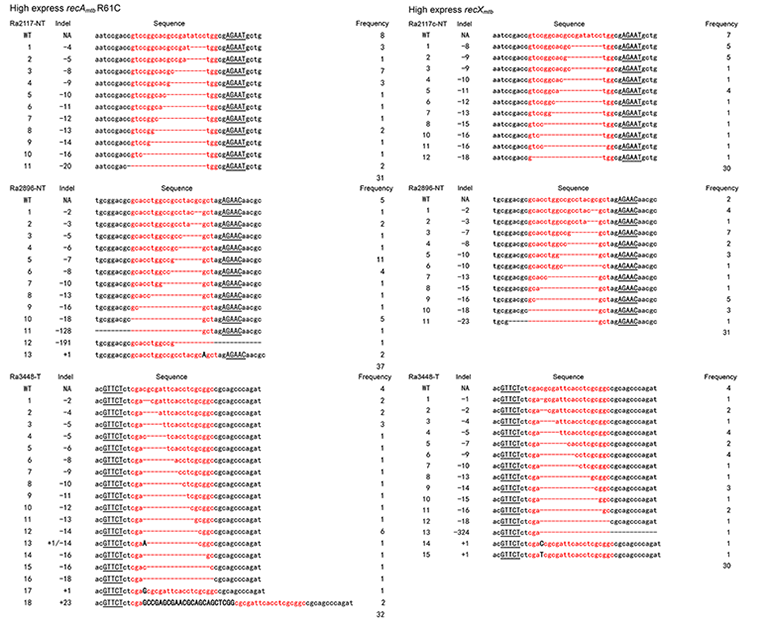

Supplement: FIG S6 [file mBio.02364-19-sf006.tif]
