# Supplementary material for: A CRISPR-Assisted Nonhomologous End-Joining Strategy for Efficient Genome Editing in Mycobacterium tuberculosis
Source: mBio. 2020 Jan 28;11(1):e02364-19. doi: 10.1128/mBio.02364-19 (PMC6989103; doi:10.1128/mBio.02364-19)
Supplement: TABLE S1 [file mBio.02364-19-st001.pdf]

Table S1

(A)

Strain	Sequence Coverage	Targeting Position	deletion size	Number of other variants[g]
M.marinum				
whiB6_1	538.3		Δ13bp	2
whiB6_2	388.1		Δ133bp	3
whiB6_3	347.4	6577131-6577155	Δ404 bp	3
whiB6_4	375.2		Δ1320 bp	3
whiB6_5	360.7		Δ9206 bp	2
whiB6_6	380.7		Δ10179 bp	2
**H37Ra**				
RecX [a]	555.2	-	-	1
vapC47_1[b]	665.2	3835504-3835524	Δ15bp	0
relG_1[b]	601.2	3189961-3189982	Δ16bp	0
vapC43_1[b]	870.1	3195700-3195720	Δ3bp	0
vapC22_1[c]	940.3	3148944-3145963	Δ15bp	1
$RecA_{mu}$[d]	467.7	-	-	3
vapC43_2[e]	503.2	3195700-3195720	Δ191bp	0
relK_1[e, f]	1580.8	3780210-3780230<br>3780235-3780254	Δ28bp	0

[a] H37Ra harboring the helper plasmid pNHEJ-*recX*.

[b] Genome editing using the helper plasmid pNHEJ-*recX* .

[c] Genome editing using the helper plasmid pNHEJ-*recX*-*sacB*.

[d] H37Ra harboring the helper plasmid pNHEJ- $recA_{mu}$.

[e] Genome editing using the helper plasmid pNHEJ-$recA_{mu}$.

[f] Paired sgRNAs were used for genome editing.

[g] The tested strains were compared to the wild-type strain or the strain harboring the corresponding helper plasmid.

(B) The SNVs or INDELs detected via whole-genome sequencing as in Table S1A.

Strain_Clone	Type	Region	Genes	References	Alleles
M.marinum					
whiB6_1/2/3/4/5/6	SNV	113853	MMAR_0099	C	G
whiB6_1/2/3/4/5/6	SNV	4013302	MMAR_3268	A	C
whiB6_2	SNV	1309419	*rpsD*	C	T
whiB6_3	SNV	6635724	MMAR_5578	C	A
whiB6_4	INDEL	3789244	intergenic	-	C
**H37Ra**					
RecX	SNV	1474556	MRA_r01	A	G
vapC22_1	INDEL	3977765	intergenic	C	-
$RecA_{mu}$	SNV	136884	MRA_0117	T	C
	SNV	1474556	MRA_r01	A	G
	SNV	3043962	lexA	G	A
