# Supplementary material for: A CRISPR-Assisted Nonhomologous End-Joining Strategy for Efficient Genome Editing in Mycobacterium tuberculosis
Source: mBio. 2020 Jan 28;11(1):e02364-19. doi: 10.1128/mBio.02364-19 (PMC6989103; doi:10.1128/mBio.02364-19)
Supplement: TABLE S2 [file mBio.02364-19-st002.pdf]

Table S2 Genome editing of regions with "weak" PAM sequences in *M. tuberculosis*.

Gene name	sgRNA targeting sequence +PAM sequence[a]	Fold repression[b]	No. of mutant / No. of test
Rv1991c	actgacctcaccgaccgagTTGGGGAG	24.7	10/16
Rv1991c	gtacggatctgactggattaCGAGCAC	11.9	16/16
Rv2801c	gtgatgcgccgcggtgagatTTGGCAG	4.0	14/24
Rv2801c	accttggcgatgttgctcgtcaCCGGCAC	2.7	19/24

[a] PAM sequences are shown underlined and capitalized.

[b] The PAM fold repression was adopted from Ref (1). A cutoff score of greater than 25-fold repression was suggested for CRISPRi; thus PAMs with less than 25-fold repression are considered "weak".

Reference

1. Rock JM, Hopkins FF, Chavez A, Diallo M, Chase MR, Gerrick ER, Pritchard JR, Church GM, Rubin EJ, Sassetti CM, Schnappinger D, Fortune SM. 2017. Programmable transcriptional repression in mycobacteria using an orthogonal CRISPR interference platform. Nat Microbiol 2:16274.
