# Supplementary material for: A CRISPR-Assisted Nonhomologous End-Joining Strategy for Efficient Genome Editing in Mycobacterium tuberculosis
Source: mBio. 2020 Jan 28;11(1):e02364-19. doi: 10.1128/mBio.02364-19 (PMC6989103; doi:10.1128/mBio.02364-19)
Supplement: TABLE S3 [file mBio.02364-19-st003.pdf]

Table S3 Genome editing in *M. tuberculosis* H37Rv and *M. bovis* BCG.

Gene name	Description	No. of mutant/ No. of test
**H37Rv**		
Rv0059	Toxin	9/12
Rv0299	Toxin, MazF10	6/8
Rv0301	Toxin, VapC2	3/3
Rv0609	Toxin, VapC28	7/8
Rv0919	Toxin	4/8
Rv2010	Toxin,VapC15	12/14
Rv1273c-1272c*	Drug ABC transporter ATP-binding protein	14/16
**BCG**		
Mb1074	Toxin	10/13
Mb2057c	Heat shock protein hspx	13/16
Mb2891	Toxin, RelG	6/10
Mb2897	Toxin, VapC43	5/8
Mb1304c-1303c*	Probable drugs-transport transmembrane ATP-binding protein ABC transporter	20/24

* Double sgRNAs were used to delete the entire operon.
