# Supplementary material for: A CRISPR-Assisted Nonhomologous End-Joining Strategy for Efficient Genome Editing in Mycobacterium tuberculosis
Source: mBio. 2020 Jan 28;11(1):e02364-19. doi: 10.1128/mBio.02364-19 (PMC6989103; doi:10.1128/mBio.02364-19)
Supplement: TABLE S4 [file mBio.02364-19-st004.pdf]

Table S4 Plasmids used in this study.

Strains	Relevant characteristic(s)	Source and or reference
pJV53-Cas12a	Codon optimized Cas12a under control of the P*myc1tetO* promoter inserted in pJV53 (*oriM*; *oriE*; Km^r; Che9c; Cas12a)	reference (1)
pCR-Hyg	Shuttle vector containing crRNA cassette under contro of *hsp60* promoter (pBR322 ori; pAL5000ts; Hyg^r)	reference (1)
pCR-Zeo	Shuttle vector containing crRNA cassette under control of *hsp60* promoter (pBR322 ori; pAL5000ts; Zeo^r)	reference (1)
pBp4	Shuttle vector containing a replicon of mycobacterial self-replicative plasmid pMF1 (pBR322 ori; pMF1 replicon; Km^r)	reference (2)
pNHEJ	*Mycobacterium smegmatis ku* (*MSMEG_5580*) and *ligaseD* (*MSMEG_5570*) gene under control of arabinose promoter from pKD46 (*oriE*; Cm^r; *ku*;*ligD*)	reference (3)
pZCas9	A plasmid carrying *Cas9* gene (p15A ori) (Kan^r) (p15A ori; Km^r; *Cas9*;)	reference (3)
psgRNA-lacZ	A plasmid with a sgRNA (*lacZ* spacer) targetting *lacZ* gene (pBR322-ori; Cm^r)	reference (3)
pLJR965	CRISPRi integrating plasmid expressing $dCas9_{sth1}$ and the cognate sgRNA (L5; *oriE*; Km^r; $dCas9_{sth1}$;)	reference (4)
pSL003	Shuttle vector expressing site-specific recombinase. (oriM; oriE; Zeo^r; Cre)	reference (5)
pMV261-Cas12a	Optimized Cas12a under control of the P_{myc1}*tetO* promoter inserted in pMV261 (*oriM*; *oriE*; Kn^r; Cas12a)	reference (1)
pYC1103	*whiB6* crRNA inserted in pCR-Hyg digested with *Bpm*I and *Hind*III (pBR322 ori; pAL5000ts;gfp crRNA cassette; Hyg^r)	This study
pYC1178	*recD* crRNA inserted in pCR-Hyg digested with *Bpm*I and *Hind*III. (pBR322 ori; pAL5000ts;gfp crRNA cassette; Hyg^r)	This study
pYC1521	*nrgA* crRNA inserted in pCR-Hyg digested with *Bpm*I and *Hind*III (pBR322 ori; pAL5000ts;gfp crRNA cassette; Hyg^r)	This study
pNHEJ-Cas12a	NHEJ machinery (Ku-NrgA-LigD) of *M. marinum* inserted into KpnI and NheI site of pJV53-Cas12a (*oriE*; *oriM*; Km^r; MmNHEJ; Cas12a)	This study
pNHEJ-Cas12a-*recX*	The *recX* from the *M. smegmatis* under control of P*myc* inserted into *Hind* III and Sal I site of pNHEJ-Cas12a (*oriE*; *oriM*; Km^r; MmNHEJ; *recX*)	This study
pNHEJ-Cas12a-$recA_{mu}$	The *recA R60C* from the *M. smegmatis* under control of native promoter inserted into *Hind* III and *Sal* I site of pNHEJ-Cas12a	This study

	(*oriE*; *oriM*; Km^r; MmNHEJ; *recA*$_{mu}$)	
pYC1376	NHEJ machinery without NrgA of *M. marinum* inserted into KpnI and NheI site of pNHEJ-Cas12a-*recX* (*oriE*; *oriM*; Km^r; *ku*; *ligD*; *recX*)	This study
pYC1655	The sgRNA under the control of an optimized TetR-regulated promoter inserted into pSL003 (pBR322 ori; pAL5000ts; Zeo^r)	This study
pYC1286	dCas9$_{sth1}$ reverted to nuclease-active Cas9 in pLJR965 (L5; *oriE*; Km^r; Cas9$_{sth1}$;)	This study
pNHEJ	pNHEJ-Cas12a digested with SpeI and religated to delete Cas12a (*oriE*; *oriM*; km^r; MmNHEJ;)	This study
pNHEJ-Cas9$_{sth1}$	Cas9$_{sth1}$ under control of the P$_{myc1}$*tetO* promoter replace Cas12a (*oriE*; *oriM*; Km^r; MmNHEJ; Cas9$_{sth1}$)	This study
pNHEJ-*recX*	The *recX* from the *M. tuberculosis* under control of P*myc* inserted into KpnI site of pNHEJ (*oriE*; *oriM*; Km^r; MmNHEJ; *recX*)	This study
pNHEJ-*recA*$_{mu}$	The *recA R61C* from the *M. tuberculosis* under control of native promoter inserted into *Kpn* I site of pNHEJ (*oriE*; *oriM*; Km^r; MmNHEJ; *recA*$_{mu}$)	This study
pYC1654	pYC1376 digested with SpeI and religated to delete Cas12a (*oriE*; *oriM*; Km^r; *ku*; *ligD*; *recX*)	This study
pYC1446	Integrating plasmid expressing Cas9$_{sth1}$ and the cognate sgRNA (L5; *oriE*; Cas9$_{sth1}$; Zeo^r)	This study
pNHEJ-*recX-sacB*	*sacB* cloned into pNHEJ -recX$_{mtb}$ (*oriE*; *oriM*; Km^r; MmNHEJ; *recX*; *sacB*)	This study
pNHEJ- *recA*$_{mu}$*-sacB*	*sacB* cloned into pNHEJ -recA$_{mu}$ (*oriE*; *oriM*; Km^r; MmNHEJ; *recX*; *sacB*)	This study
pYC1640	Shuttle vector containing cas9$_{sth1}$,sgRNA cassette and the pMF1 replicon. (*oriE*; pMF1 replicon; Zeo^r)	This study
pYC2085	Shuttle vector containing cas9$_{sth1}$, sgRNA cassette and the pMF1 replicon. (*oriE*; pMF1 replicon; Hyg^r)	This study
pZCas9-*recX*	The *recX* from the *E.coli* under control of native promoter inserted into pZCas9 (p15A ori; Km^r; *Cas9*; *recX*)	This study

References

1. Yan M-Y, Yan H-Q, Ren G-X, Zhao J-P, Guo X-P, Sun Y-C. 2017. CRISPR-Cas12a–assisted recombineering in bacteria. Applied and Environmental Microbiology doi:10.1128/aem.00947-17.
2. Bachrach G, Colston MJ, Bercovier H, Bar-Nir D, Anderson C, Papavinasasundaram KG. 2000. A new single-copy mycobacterial plasmid, pMF1, from Mycobacterium fortuitum which is compatible with the pAL5000 replicon. Microbiology 146:297-303.
3. Zheng X, Li SY, Zhao GP, Wang J. 2017. An efficient system for deletion of large DNA fragments in Escherichia coli via introduction of both Cas9 and the non-homologous end joining system from Mycobacterium smegmatis. Biochem Biophys Res Commun 485:768-774.
4. Rock JM, Hopkins FF, Chavez A, Diallo M, Chase MR, Gerrick ER, Pritchard JR, Church GM, Rubin EJ, Sassetti CM, Schnappinger D, Fortune SM. 2017. Programmable transcriptional repression in mycobacteria using an orthogonal CRISPR interference platform. Nat Microbiol 2:16274.
5. Wu X, Fang W, Yu Y, Song H. 2012. [Development and application of homologous recombination knockout system in Mycobacterium tuberculosis]. Wei Sheng Wu Xue Bao 52:1151-9.
